# A Versatile SAW Sensor-Based Modular and Portable Platform for a Multi-Sensor Device

**DOI:** 10.3390/mi16020170

**Published:** 2025-01-31

**Authors:** Ángel López-Luna, Patricia Arroyo, Daniel Matatagui, Carlos Sánchez-Vicente, Jesús Lozano

**Affiliations:** 1 Industrial Engineering School, University of Extremadura, 06006 Badajoz, Spain; angellopez@unex.es (Á.L.-L.); parroyoz@unex.es (P.A.); 2 Instituto de Magnetismo Aplicado (IMA), Universidad Complutense de Madrid-Administrador de Infraestructuras Ferroviarias (UCM-ADIF), A-6, 900, 28232 Las Rozas, Spain; daniel.m.c@ucm.es; 3 Departamento de Física de Materiales, Universidad Complutense de Madrid (UCM), Plaza de las Ciencias 1, 28040 Madrid, Spain; 4 Institute for Physical and Information Technologies (ITEFI), CSIC, Serrano 144, 28006 Madrid, Spain; c.sanchez.vicente@csic.es

**Keywords:** electronic nose, SAW, heterodyne system

## Abstract

This study presents the development and characterization of a novel electronic nose system based on customized surface acoustic wave (SAW) sensors. The system includes four sensors, customized with different custom polymer coatings, in order to detect volatile organic compounds (VOCs). The main innovation lies in the design of a robust and versatile switching electronics system that allows for the integration of the SAW sensors into portable systems, as well as interoperability with other gas sensor technologies. The system includes a modular architecture that allows multiple sensor arrays to be combined to improve the selectivity and discrimination of complex gas mixtures. To verify the proper performance of the system and the detection capability of the manufactured sensors, experimental laboratory tests have been carried out. Specifically, ethanol and acetone measurements up to a 2000 ppm concentration have been performed. These preliminary experimental results demonstrate the capability of the SAW sensors with different response patterns across the sensor array. In particular, the sensor made with the polyvinyl acetate polymer exhibits high sensitivity to both VOCs.

## 1. Introduction

Surface acoustic wave (SAW) gas sensors have been studied in recent years due to their sensitivity and versatility in detecting various gases and volatile compounds. Specifically, they exhibit high sensitivity for detecting biogases, chemical agents, and volatiles in general [1]. The operation of these sensors is based on the interaction between surface acoustic waves and the analyzed medium, where changes in the properties of the medium, such as mass, temperature or viscosity, affect the frequency of the wave. Consequently, as the medium changes, the presence of gases can be detected. This technology makes SAW sensors suitable for use in environmental monitoring [1,2,3,4,5], food safety [6,7,8] and healthcare applications [9,10,11,12], among others [3,13,14].

Therefore, the incorporation of SAW sensors into electronic noses (e-noses) is an effective technique for real-time gas detection. E-noses are devices that replicate the human olfactory system and are based on sensor arrays, electronic instrumentation, and classification algorithms to detect and analyze various gases.

Recent research has focused on improving the performance of these sensors by modifying surface coatings deposited on the piezoelectric substrate, which are essential in the detection of specific gases. Metal oxide semiconductors, carbon-based materials and polymers have been used as sensitive layers. The development of nanostructured materials and the improvement of existing ones have contributed to improving the selectivity and sensitivity of the sensors [15,16].

On the other hand, this technology presents other limitations and challenges that are not directly related to the materials used in sensor fabrication. SAW sensors require complex circuitry that is temperature-sensitive. Furthermore, the integration of multiple sensors remains a challenge, as does the optimization of signal-processing techniques for these sensors. Research and development in this area would open the door to integrating these sensors into portable and modular devices. This, combined with advances in Internet of Things (IoT) technology, would enable the creation of a network of devices with different detection capabilities based on the target gaseous compounds to be measured.

This study aims to address some challenges by designing a portable e-nose system utilizing SAW sensors that work with Love waves. Thus, the present work presents a novel e-nose system that incorporates customized SAW sensors and features a robust electronic design with a switch system. This electronic system was specifically developed to maximize the functionality of SAW sensors, enabling their integration with other e-nose systems. In this way, it facilitates progress towards hybrid solutions that combine various sensing technologies to optimize performance.

This paper is structured as follows. First, Section 2 details the fabrication of the SAW sensors, the electronic system designed and developed, and the experimental setup used for sensor characterization. Section 3 focuses on the results obtained in the detection of the studied VOCs (ethanol and acetone)and are discussed.Finally, Section 4 summarizes the main contributions of this work and possible future work.

## 2. Materials and Methods

### 2.1. SAW Sensor Manufacturing

Customized SAW sensors were developed as the main sensing element of the electronic nose. For this purpose, substrates developed in previous work were used [17,18]. These sensors are compact, facilitating integration into portable systems, such as the one presented in this work. Additionally, they can operate in both gaseous and liquid phases and can be adapted to detect specific gases depending on the materials used in their manufacture. Moreover, they can operate at ambient temperature.

A key factor influencing the sensitivity of the sensor is its operating frequency, which, in this study, was set at approximately 168 MHz. For the development of the sensors, four polymer solutions were prepared and applied to the quartz substrate of the SAW sensors. An appropriate organic solvent was used to dissolve each of the polymers. The material application process involved continuous monitoring of the sensor’s operating frequency. The prepared solution was poured into an airbrush and the sensor was coated with the polymer solution using compressed nitrogen until a frequency shift of approximately 300 kHz was achieved. Table 1 provides the characterization of the sensors, obtained using a Keysight N5230A (Agilent, Santa Rosa, CA, USA) network analyzer along with the polymers used in the fabrication of each sensor. Table 2 shows the proportions and solvents used in each solution.

It should be noted that the SAW 3 sensor was found to have a manufacturing defect and provided a lower gain than the other sensors.

The polymer materials selected for this work are compounds that were previously used in the research group, with which there is prior experience. Additionally, the thin film deposition process with these materials is simple, fast, and economical.

### 2.2. Description of the Prototype

#### 2.2.1. Electronic Nose Description

The electronic nose developed in this study is based on the use of the SAW sensors described in Section 2.2, together with an electronic circuit specifically designed to process the signals they generate. Specifically, the switched circuit with SAW sensors is the main focus of this study. The system comprises five SAW sensors: four are coated with different polymers for gas detection, while the fifth serves as a reference sensor.

Each SAW sensor is equipped with an oscillator circuit that includes two MAR-8ASM+ monolithic amplifiers from Mini-Circuits (Brooklyn, NY, USA). The circuit features a 4th-order Chebyshev bandpass filter, which is tuned to the oscillation frequency, along with passive attenuators that stabilize the amplitude. A coupler is incorporated to isolate the circuit from the other electronics, which helps to improve the robustness of the system against possible interference and fluctuations in the rest of the electronic environment. The oscillation of the system is initiated by the inherent thermal noise of the components, which is then amplified and filtered to produce a stable signal. Additionally, the circuit is designed to compensate for gain loss between sensor ports, as indicated in the gain column of Table 1.

The outputs of the couplers are routed to an ADG904BRUZ switcher for RF signals from Analog Devices (Wilmington, MA, USA), which switches the signal to an ADEX-10H+ frequency mixer from Mini-Circuits. This mixer also receives the reference SAW signal, which was further amplified to achieve a higher amplitude. Within the mixer, a heterodyne frequency is generated.

This mixed signal is filtered through a low-pass filter and subsequently sampled using a 32-bit STM32WB55CC microcontroller from STMicroelectronics (Geneva, Switzerland). This microcontroller has two cores: the main core is an ARM Cortex-M4 configured at 32 MHz, and the secondary core is an ARM Cortex-M0+ also at 32 MHz, dedicated exclusively to Bluetooth communication. It is equipped with 1 Mbyte of flash memory, two comparators, BLE 5.4, two SPI buses, two I^2^C interfaces, two DMA channels, and six timers, including two low-power timers, among other peripherals.

Therefore, the microcontroller is responsible for switching the RF signals through two digital signals sent to the ADG904BRUZ, obtaining the frequency of the heterodyne signal from the mixer, communicating it to an external computer via UART, and managing connections to other electronic nose PCBs.

**Figure 1:**
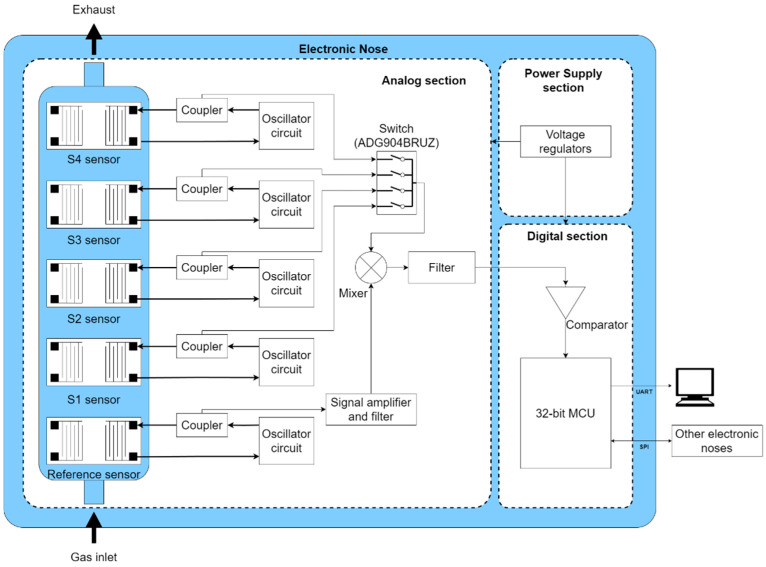
Diagram of the electronic nose design utilizing SAW sensors.

The whole system is powered by a 9 Vdc input for the oscillator circuits, adapted to 3.3 Vdc for the control electronics using two voltage regulators, the LD1117S50TR and the LD39050PU33R, both from STMicroelectronics. Figure 1 illustrates the schematic of the electronic nose system and its operation.

**Figure 2:**
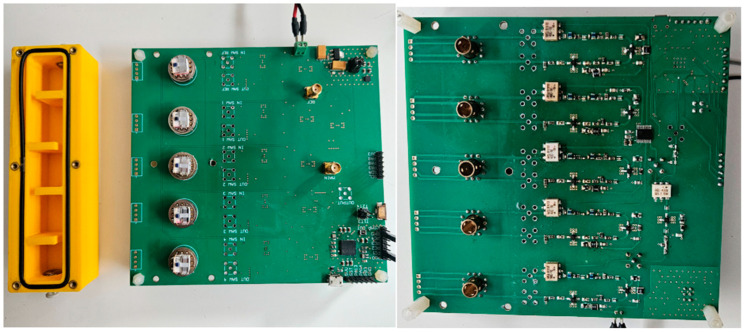
On the right is the bottom layer of the PCB. On the left is the top layer next to the gas cell.

A custom-designed printed circuit board (PCB) was developed to host the electronic circuit described. The PCB was designed using Altium Designer Professional (version 23.11) and manufactured by JLCPCB (Hong Kong, China). Portability was a key factor in the design to allow the eNose to be used in future field applications, with final PCB dimensions of 140 × 132 mm. SMA connectors were included to facilitate the verification of sensor signals using a Rohde & Schwarz MXO 4 oscilloscope and a Keysight N5230A network analyzer. Figure 2 presents the PCB with all electronic components assembled.

#### 2.2.2. Operating Algorithm

The algorithm implemented in the microcontroller works by counting the pulses of the signal generated in the mixer, which obtains the frequency difference between the SAW sensor and the reference, providing the absolute value of this difference.

To achieve this, the microcontroller resets an internal counter, switches the output to select the sensor to be measured, and initiates a 1-second timer. During this period, the counter records the positive half-cycles of the signal. After the one-second interval, the microcontroller calculates the signal frequency and transmits a character string via the UART interface to a computer. This string specifies the active sensor and the measured frequency. The precision of the measurement is ensured by sampling the signal at a rate considerably higher (approximately 10.6 times) than the signal frequency, following the Nyquist–Shannon theorem.

Furthermore, the microcontroller can control an external solenoid valve S070C-RAG-32 from SMC (Chiyoda, Tokyo, Japan) using a digital signal to automate air sampling, allowing for alternation between absorption and desorption periods. This functionality enhances sensor regeneration and supports cyclic measurements.

#### 2.2.3. Gas Cell Design

For the characterization of SAW sensors, a gas cell was designed to facilitate exposure to gas samples. The cell was modeled using Autodesk Inventor Professional 2024 software and fabricated via 3D printing with an Artillery Sidewinder X4 Plus printer. The printing material chosen was Colorfabb nGen co-polyester, selected for its odorless properties, which minimize interference with sensor measurements. The filament used had a diameter of 1.75 mm. The dimensions of the gas cell are 132 × 39 × 24 mm, featuring a serpentine circuit with five cavities measuring 20 × 20 mm to accommodate each SAW sensor. Gas is introduced into the cell through a nylon tube with an internal diameter of 3 mm.

#### 2.2.4. Interoperability with Other Electronic Noses

To increase the versatility and analytical capability of the electronic nose, the system incorporates a modular design capable of interfacing with other detection systems. Specifically, it supports communication with TOMATO-NOSE and NEONOSE, devices previously developed by the research group [19,20]. This integration is achieved through an SPI (Serial Peripheral Interface) bus, which enables communication between the microcontrollers present in each eNose by transferring data packets between them.

The interconnection with these other olfactory and sensing systems offers the possibility to combine multiple sensor technologies (digital MOS, NDIR, PID, and electrochemical) to improve the system’s ability to discriminate between various gases. The modular architecture allows the construction of a stack with two interchangeable boards with a gas cell in between, each equipped with different types of sensors facing the cell, making it possible to configure the system to meet the specific requirements of each application. This hybrid approach optimizes both selectivity and sensitivity to the gases of interest.

### 2.3. Experimental Procedure

In order to evaluate the performance of the electronic nose, experiments were conducted where specific gas samples were introduced into the sensor cell at a constant flow rate. For each gas mixture, sampling cycles were performed with a duration of 2 min, interspersed with desorption periods of 2 min.

During each sampling cycle, the response of the four SAW sensors was recorded at a frequency of 1 Hz per sensor, meaning one measurement was taken per sensor every 4 s. This procedure enabled the acquisition of time-series data for each sensor and each gas concentration, facilitating the analysis of the dynamic response of the electronic nose.

#### 2.3.1. Samples

Ethanol and acetone vapors were selected for this study. These compounds were selected due to their ease of procurement and their classification as volatile organic compounds (VOCs) with a strong characteristic odor. These compounds were tested at two concentrations each: 921.1 ppm and 1991 ppm for acetone, and 976.1 ppm and 1994.5 ppm for ethanol. To achieve these concentrations, the static headspace technique was used, which facilitated air and analyte mixing under controlled conditions. For this purpose, 20 mL of each sample was prepared in vials that were kept at 30 °C in a thermal bath. The established headspace generation time was 20 min. The concentration of the saturated headspace of an analyte was determined using Dalton’s Law of Partial Pressures and the Antoine equation, whose coefficients can be found in established publications [21], dependent on both the chemical compound and temperature. The prepared gas mixtures for the experiment are presented in Table 3, which includes the vapor pressure at the experimental temperature (P_i-pure_), density, mole fractions of water (X_water_) and the compound (X_i_), the partial pressure of the compound in the headspace (P_i_), and the concentration in parts per million (C).

#### 2.3.2. Measurement Setup

**Figure 3:**
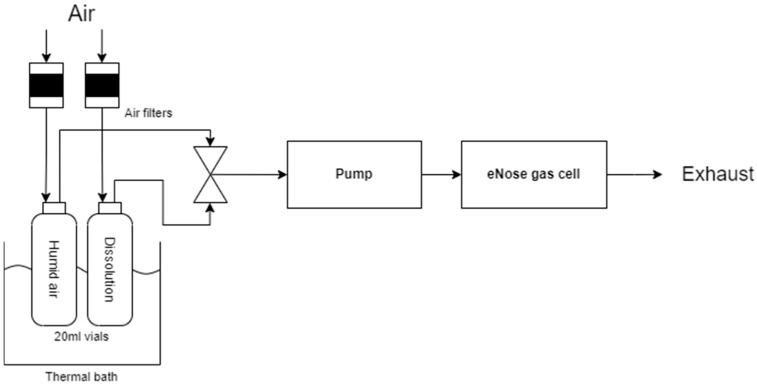
Diagram of the experiment carried out.

Figure 3 illustrates the schematic of the measurement setup employed for the characterization of the electronic nose. A thermal bath set at 30 °C was utilized to maintain a constant temperature for the two vials containing the samples: one with the volatile organic compound (ethanol or acetone) dissolved in water, and the other containing only water. The presence of water in both vials enabled the creation of a headspace with equivalent relative humidity in both cases, thereby minimizing the interference of humidity in the sensor measurements.

Air was pumped through the system at a controlled flow rate of 130 mL/min, directing it through two filters that removed any suspended particles to ensure that the air was pure. The air then flowed into the gas cell, where the five SAW sensors were housed. A solenoid valve was employed to alternate between air from the vial containing the volatile organic compound solution and air from the vial with water. This allowed for switching between sampling and desorption cycles. Finally, the air, after passing through the gas cell, was expelled into the atmosphere. This experimental setup was designed to ensure the reproducibility of measurements and to minimize the influence of external factors, such as temperature and humidity, on the sensor response. Three complete measurement cycles were performed for each ethanol and acetone concentration.

## 3. Results and Discussion

### 3.1. Laboratory Test Results

**Figure 4:**
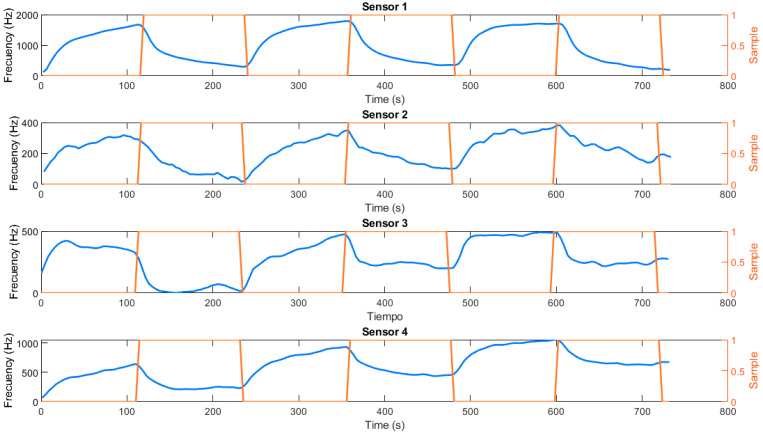
Temporal curves of the sensors during exposure to 1994.5 ppm ethanol cycles.

During the execution of all the measurements, a script executed in Matlab R2023a receives the data transmitted by the microcontroller through the USB port of the computer. The software performs basic preprocessing, graphs, and stores the data for further analysis. First, it must be taken into account that the response dynamics depend on whether each sensor’s resonance frequency is above or below the reference SAW sensor’s frequency (163.5 Hz). As shown in Table 1, SAW1 and SAW2 sensors have higher frequencies, resulting in an increased response upon contact with the measured VOCs, while SAW3 and SAW4 exhibit decreased responses. To improve visualization, the curves of SAW1 and SAW2 were inverted. Additionally, the minimum value of each sensor signal was subtracted in order to visualize temporal curves, as the frequency variation is very low compared to heterodyne frequencies. An example of the obtained curves can be visualized in Figure 4. It represents the temporal dynamics of the four sensors during exposure to ethanol vapor at 1994.5 ppm and subsequent desorption cycles (exposure to filtered air). A binary signal can be seen in red (sample), which takes a value of 0 in the desorption phases and 1 in the adsorption phases.

During the ethanol exposure phase, all sensors display a rapid decrease in response frequencies. In particular, sensor SAW1 experiences the largest frequency shift, suggesting a strong interaction with ethanol, probably due to the polarity of its polymer coating (PVA). Sensor SAW4 (PEG) also shows a pronounced response, consistent with its ability to form hydrogen bonds with ethanol molecules. Sensors SAW2 (PS) and SAW3 (PDMS), however, exhibit smaller displacements, indicating less interaction with ethanol. In addition, this low response leads to noise appearing in the signal. During the filtered air exposure phase, all sensors show a gradual recovery towards the reference frequencies. This recovery highlights the reversibility of the adsorption process, especially in the case of sensors SAW1 and SAW4. The SAW1 sensor maintains slower desorption, potentially due to stronger bond interactions with ethanol. However, it is quite repeatable and no drift effect is observed in the sensor. In contrast, the SAW3 sensor shows faster recovery, but also higher drift. Finally, it should be noted that the SAW4 sensor appears to experience large short-term drift.

**Figure 5:**
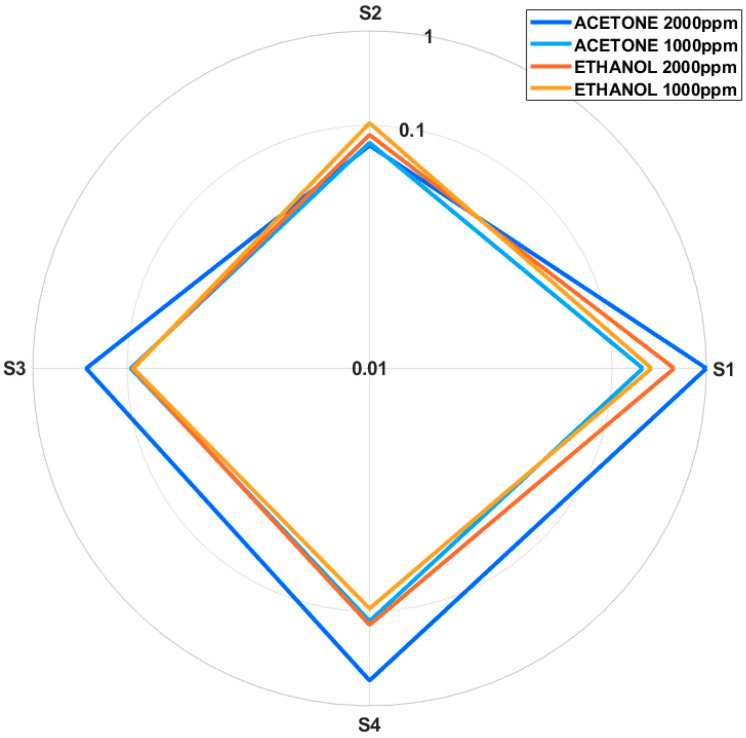
Radial plot of normalized sensor responses in logarithmic scale.

On the other hand, the response of each sensor in each measurement cycle has been calculated. For this purpose, an average of the maximum values reached during the desorption cycle and the average of the minimum values reached during the adsorption cycle were calculated. The difference between the two values was considered the response. Figure 5 shows a radial diagram of the responses obtained by each sensor. The radial diagram, in logarithmic scale, illustrates the responses of the SAW sensors to ethanol and acetone at different concentrations, providing a detailed view of their sensitivity and selectivity profiles. In it, each axis represents a specific sensor. Thus, variations in response as a function of the polymer coatings applied are revealed. The logarithmic scale of the graph is used to achieve a clearer visualization of the differences in response, particularly for lower concentrations.

It can be seen that the sensors have concentration-dependent responses for both ethanol and acetone. The higher concentrations generally produce higher responses in all sensors. For acetone at 2000 ppm, the SAW1 sensor shows the highest response, with a normalized value of 1, which could indicate a strong affinity between acetone molecules and the polar PVA coating. The SAW4 sensor follows with a significant response, as does the SAW3 sensor. The response of the SAW2 sensor is noticeably lower, reflecting a weaker interaction perhaps due to the hydrophobic or semi-polar nature of its coating. At 1000 ppm acetone, the sensors show smaller responses, with Sensor SAW1 maintaining the highest value, followed by Sensor SAW4 and SAW3, while Sensor SAW2 barely reduces its response.

In the case of ethanol, the same trend is observed: SAW1 shows the strongest response, followed by SAW4, SAW3 and SAW2. However, the relative differences between the sensor responses vary with concentration. At 2000 ppm, the difference between SAW1 and SAW4 is more significant than for acetone. At 1000 ppm, the responses decrease proportionally for SAW1 and SAW4, with minimal changes for SAW3, which could be saturated for the study concentrations. However, SAW2 shows an unexpected increase in its response, showing incorrect functionality for this application.

These results highlight the complementary behavior of the set of sensors. SAW1 consistently exhibits the highest sensitivity for both VOCs, especially at higher concentrations, playing a key role in the overall detection capability of the system. The SAW4 (PEG) sensor demonstrates versatility for both analytes, while the SAW3 (PDMS) sensor enhances the system’s ability to discriminate between different gases. The SAW2 sensor does not seem to perform properly for this application; so, its incorporation in systems for the detection of these two VOCs should be considered. Additionally, a possible saturation of the SAW3 sensor is detected and future studies will be carried out to evaluate the saturation limits of the coatings used for different concentrations.

### 3.2. Statistical Analysis of Sensor Responses

#### 3.2.1. Repeatability and Variance Analysis

In order to evaluate the robustness and repeatability of the sensor responses, a statistical analysis was performed based on the calculated responses of each measurement (non-normalized). The coefficient of variation (CV) was calculated for each sensor and concentration. In addition, an analysis of variance (ANOVA) was performed to determine the statistically significant differences in the sensor responses. CV was used to evaluate the relative dispersion of sensor responses. SAW1 showed a consistent performance, with CV values ranging from 5.06% to 10.02%, showing the highest stability. Similarly, SAW4 also appeared to show good reproducibility for ethanol, with CV values below 10%, although with a worse performance in the detection of acetone. On the other hand, SAW3 obtains values below 10% at the highest concentrations; however, the variability increases at lower concentrations up to almost 18%, highlighting the need for further refinement. Finally, SAW2 shows a high variability with 1000ppm acetone (33.68%), reflecting limited repeatability under these conditions. It achieved acceptable CV values for the rest of the measurements. 

The summarized statistical parameters, including the mean frequency shift (Δf), standard deviation (σ), and coefficient of variation (CV) for each sensor and concentration, are presented in Table 4.

Since this study is preliminary, the number of repetitions per concentration was limited. Although the CV values provide a useful initial assessment of repeatability, additional repetitions would allow for a more robust analysis. Nevertheless, the results show the potential of the system, in particular for SAW1 and SAW4, and lay the foundation for future improvements in sensor performance. 

#### 3.2.2. ANOVA Results

On the other hand, the ANOVA revealed statistically significant differences (α = 0.05) in the responses of all sensors for different concentrations of ethanol and acetone. This could confirm the ability of the sensor system to discriminate between different concentrations and compounds. 

Moreover, the F-statistic obtained in the ANOVA quantifies the ratio of variability between concentrations to variability within concentrations. A higher F indicates that the differences between the responses at various concentrations are much larger than the inherent variability in the sensor’s measurements. The results of the test confirm significant differences in sensor responses across the studied concentrations:

- SAW1: F = 138.17, *p*-value = 3.13 × 10^−7^. The high F-value indicates that the differences between concentrations for this sensor are highly significant, confirming its robust and consistent performance.
- SAW2: F = 7.77, *p*-value = 0.009. Although significant, the lower F-value compared to that of other sensors reflects the limited sensitivity and higher noise observed for this sensor.
- SAW3: F = 97.97, *p*-value = 1.2 × 10^−6^. This F-value indicates clear differences between responses at various concentrations, although the variability within the measurements remains higher than for SAW1 and SAW4.
- SAW4: F = 108.78, *p*-value = 7.98 × 10^−7^. This sensor exhibits a strong ability to differentiate between concentrations, though with slightly more variability than SAW1.

These results confirm that the system is capable of providing reproducible and reliable responses, particularly for sensors SAW1 and SAW4, which exhibited the lowest variability and the highest sensitivity. The lower F-value and higher variability in SAW2 underscore the poor performance of this sensor for this application. 

## 4. Conclusions

This work presents the design and development of an electronic nose based on customized surface acoustic wave (SAW) sensors. The system design integrates four sensors with different polymer coatings. The novelty lies in the switching electronics and the modular architecture, providing a robust and versatile platform that allows for seamless integration with other detection technologies and portable applications. The results allowed us to verify that the electronics and the data acquisition system are functioning correctly, in addition to studying the behavior of the sensor array. The SAW1 sensor (PVA coating) showed the highest sensitivity to both ethanol and acetone, making it essential for the overall performance of the system. The SAW4 sensor (PEG coating) showed complementary versatility, with strong responses to both VOCs, particularly to acetone. In contrast, the SAW3 sensor (PDMS coating) showed moderate sensitivity, contributing to the discrimination of analytes, while the SAW2 sensor (PS coating) showed weaker responses, with significant noise, suggesting limited applicability for the detection of ethanol and acetone. Moreover, the different aggregations in the radial plot suggest that the electronic nose system could effectively classify ethanol and acetone based on the combined output of the sensor array. Statistical analysis confirms the reproducibility and reliability of the system, with SAW1 and SAW4 sensors standing out for their high sensitivity and low variability, while the limited performance of SAW2 suggests the need for optimization for this application. In addition, the ability of SAW sensors to detect at a known temperature (30 °C), without the need for additional sample conditioning, makes the system a promising solution for portable real-time monitoring of VOCs in environmental or industrial settings. However, for implementation in non-laboratory conditions, future research could focus on evaluating the system’s performance over a wider temperature range (15–40 °C), developing thermal compensation algorithms, and exploring coatings that are less sensitive to temperature variations, thus ensuring greater stability and accuracy in uncontrolled environments.

## Figures and Tables

**Table 1:** Characterization of SAW sensors.

Sensor	Oscillation Frequency Without Polymer	Gain	Applied Polymer	Δf with Polymer
Reference SAW	163.95 MHz	−22 dB	None	-
SAW 1	166.4 MHz	−22 dB	Polyvinyl acetate (PVA)	−300 KHz
SAW 2	165 MHz	−21.5 dB	Polystyrene (PS)	−300 KHz
SAW 3	163 MHz	−47.753 dB	Polydimethylsiloxane (PDMS)	+300 KHz
SAW4	163 MHz	−21 dB	Polyethylene glycol (PEG)	+300 KHz

**Table 2:** Prepared solutions for the sensitive film of the SAW sensors.

Solution	Polymer (g)	Solvent (mL)	Polymer Brand	Polymer Catalog Number
PVA + H_2_O	0.04023	20.1	Sigma-Aldrich	341584
PDMS + dichloromethane	0.0423	21.32	Sigma-Aldrich	9016006
PS + xylene	0.0384	19.2	Sigma-Aldrich	430102
PEG + dichloromethane	0.03857	19.2	Dow Chemical	Carbowax-1000

**Table 3:** The solutions prepared for testing the functionality of the electronic nose.

Compound	Molecular Weight	P_i-pure_	Density (g/cm^3^)	X_water_	X_i_	P_i_ (kPa)	C (ppm)
Acetone (921.1 ppm)	58.0791	37.82	0.790	0.550	0.0014	0.0933	921.1
Acetone (1991 ppm)	58.0791	37.82	0.790	0.761	0.0041	0.20169	1991
Ethanol (976.1 ppm)	46.0684	10.47	0.789	0.538	0.0051	0.09888	976.1
Ethanol (1994.5 ppm)	46.0684	10.47	0.789	0.522	0.0103	0.20205	1994.5

**Table 4:** Data from the statistical analysis of the laboratory test.

Sensor	Concentration (ppm)	Mean (Δf)	Standard Deviation (σ)	CV (%)
SAW1	2000 (Acetone)	3179.67	318.51	10.02
	1000 (Acetone)	721.67	43.82	6.07
	2000 (Ethanol)	1470.67	74.45	5.06
	1000 (Ethanol)	820.33	58.59	7.14
SAW2	2000 (Acetone)	204.67	12.66	6.19
	1000 (Acetone)	218.67	73.64	33.68
	2000 (Ethanol)	264.67	22.30	8.43
	1000 (Ethanol)	350.67	24.91	7.10
SAW3	2000 (Acetone)	883.67	84.36	9.55
	1000 (Acetone)	253.00	43.55	17.22
	2000 (Ethanol)	292.67	28.11	9.61
	1000 (Ethanol)	283.67	39.02	13.75
SAW4	2000 (Acetone)	1736.33	207.07	11.93
	1000 (Acetone)	398.33	74.27	18.65
	2000 (Ethanol)	458.67	41.48	9.04
	1000 (Ethanol)	310.00	19.00	6.13

## Data Availability

Data of sensors will be available upon request.
